# DEVELOPMENT OF A HOPE-BASED HEALTH PROMOTION PROGRAM TO IMPROVE HEALTH BEHAVIORS AMONG DIVERSE OLDER ADULTS

**DOI:** 10.1093/geroni/igac059.2031

**Published:** 2022-12-20

**Authors:** Britteny Howell, Amber Worthington, Leslie Redmond

**Affiliations:** University of Alaska Anchorage, Anchorage, Alaska, United States; UNIVERSITY OF ALASKA ANCHORAGE, Anchorage, Alaska, United States; University of Manitoba, Anchorage, Alaska, United States

## Abstract

Studies have shown that health education can improve dietary intake, exercise, and energy balance, which improves health outcomes and quality of life. However, diverse low-income older adults may have low self-efficacy coupled with potentially negative opinions regarding the aging process. Emerging research suggests that asset-based approaches utilizing persuasive and hopeful messages may be particularly effective with diverse populations of older adults who may be struggling with negative perceptions of self-efficacy. Research also shows that health behavior change interventions are most likely to be successful among diverse populations when they incorporate messages of resilience and facilitate information sharing and diffusion among participants. This presentation outlines the program curriculum development process of a student-led health promotion program by an interdisciplinary team of university researchers. Public health, dietetics, and communication faculty utilized Persuasive Hope Theory (PHT) and Self-Efficacy Theory to create a 15-week program to improve fruit and vegetable intake and physical activity among low-income adults aged 55+ living in Anchorage, Alaska. We will also detail the curricular components for this NIA-funded program which will be delivered in Fall 2022.

